# Population substructure in Cache County, Utah: the Cache County study

**DOI:** 10.1186/1471-2105-15-S7-S8

**Published:** 2014-05-28

**Authors:** Aaron R Sharp, Perry G Ridge, Matthew H Bailey, Kevin L Boehme, Maria C Norton, JoAnn T Tschanz, Ronald G Munger, Christopher D Corcoran, John SK Kauwe, Alzheimer's Disease Neuroimaging Initiative (ADNI)

**Affiliations:** 1 Department of Biology, Brigham Young University, Provo, Utah, USA; 2 Department of Family Consumer and Human Development, Utah State University, Logan, Utah, USA; 3 Department of Psychology, Utah State University, Logan, Utah, USA; 4 Department of Nutrition, Dietetics, and Food Sciences, Utah State University, Logan, Utah, USA; 5 Department of Mathematics and Statistics, Utah State University, Logan, Utah, USA; 6 Center for Epidemiologic Studies, Utah State University, Logan, Utah, USA; 7 UCLA, LA, USA

## Abstract

**Background:**

Population stratification is a key concern for genetic association analyses. In addition, extreme homogeneity of ethnic origins of a population can make it difficult to interpret how genetic associations in that population may translate into other populations. Here we have evaluated the genetic substructure of samples from the Cache County study relative to the HapMap Reference populations and data from the Alzheimer's Disease Neuroimaging Initiative (ADNI).

**Results:**

Our findings show that the Cache County study is similar in ethnic diversity to the self-reported "Whites" in the ADNI sample and less homogenous than the HapMap CEU population.

**Conclusions:**

We conclude that the Cache County study is genetically representative of the general European American population in the USA and is an appropriate population for conducting broadly applicable genetic studies.

## Background

Cryptic population differences due to heterogeneity have confounding effects on association studies, especially when analyzing complex traits and gene-gene interactions. It is also possible that genetic associations that are observed in very homogeneous populations may not be generalizable to other populations.

The Cache County study is a large longitudinal cohort study of memory, health, and aging that was initiated in 1994. This sample of 5,092 individuals represents approximately 90% of the Cache County population aged 65 and older at that time. These data are a valuable resource in genetic studies of Alzheimer's disease (AD), as well as otherdiscussion forms of dementia [1]. The founding populations and migrations to Utah by early members of the Church of Jesus Christ of Latter Day Saints have been studied extensively[2-4]. These studies have concluded that due to the large founding population, high rates of gene flow and diversity of source populations the Utah population has allele frequencies that are quite similar to the general European American population in the United States. As it was collected from one county in Utah, there have been questions as to how the genetic diversity in the Cache County sample compares to that of more broadly collected European American samples and whether that diversity affects the validity or generalizability of results obtained from the Cache County study.

The Cache County study is a large longitudinal cohort study of memory, health, and aging that was initiated in 1994. This sample of 5,092 individuals represents approximately 90% of the Cache County population aged 65 and older at that time. These data are a valuable resource in genetic studies of Alzheimer's disease (AD), as well as other forms of dementia [1]. The founding populations and migrations to Utah by early members of the Church of Jesus Christ of Latter Day Saints have been studied extensively[2-4]. These studies have concluded that due to the large founding population, high rates of gene flow and diversity of source populations the Utah population has allele frequencies that are quite similar to the general European American population in the United States. As it was collected from one county in Utah, there have been questions as to how the genetic diversity in the Cache County sample compares to that of more broadly collected European American samples and whether that diversity affects the validity or generalizability of results obtained from the Cache County study.

The purpose of this study is to compare the genetic structure of the Cache County study, which was collected from one county in northern Utah, to that of the Alzheimer's Disease Neuroimaging Initiative, a sample that was collected from several sites around the USA, and data from several other populations from the International HapMap Project.

## Methods

### Sample collection and genotyping

The Cache County study originally included 5,092 permanent residents of Cache County over the age of 64. Details of collection methods and demographics of the Cache County samples have been reported previously [1]. Briefly, samples underwent four triennial waves of data collection in a multi-stage dementia screening and assessment protocol. DNA was obtained from blood and buccal swabs as described by Breitner et al. [1] All study procedures were approved by the Institutional Review Boards of Utah State, Duke and Johns Hopkins universitys. DNA from 506 Cache County study participants was genotyped using the Illumina OmniExpress chip; DNA from 234 others was genotyped using the Illumina 2.5M chip.

The broader population data with which we will compare the Cache data was collected by The International HapMap Project [5] and the Alzheimer's Disease Neuroimaging Initiative (ADNI) [6]. The International Hapmap Project (phase 3, draft release 2) consists of SNP genotyping data from the populations in Table 1[5]. The data were collected using two genotyping platforms: Illumina Human1M by the Wellcome Trust Sanger Institute and Affymetrix SNP 6.0 by the Broad Institute. They are available for download from http://hapmap.ncbi.nlm.nih.gov/downloads/genotypes/2009-01_phaseIII/plink_format.

**Table 1:** HapMap populations with Abbreviations

Population	Abbreviation
African ancestry in Southwest USA	ASW
CEPH (Utah residents with ancestry from northern and western Europe)	CEU
Han Chinese in Beijing, China	CHB
Chinese in Metropolitan Denver, Colorado	CHD
Gujarati Indians in Houston, Texas	GIH
Japanese in Tokyo, Japan	JPT
Luhya in Webuye, Kenya	LWK
Mexican ancestry in Los Angeles, California	MEX
Maasai in Kinyawa, Kenya	MKK
Tuscan in Italy	TSI
Yoruban in Ibadan, Nigeria	YRI

The ADNI samples are part of a longitudinal study designed to measure the progression of mild cognitive impairment (MCI) and early AD [6]. The primary goal of ADNI has been to test whether serial magnetic resonance imaging (MRI), PET, other biological markers, and clinical and neuropsychological assessment can be combined to measure the progression of MCI and early AD (PI: Michael W. Weiner, M.D., VA Medical Center and University of California - San Francisco). ADNI is the result of efforts of many co- investigators from a broad range of academic institutions and private corporations, and subjects have been recruited from over 50 sites across the U.S. and Canada. For up-to-date information, see http://www.adni-info.org. Data for the present analysis were downloaded from the ADNI web site in March 2013. We used self-reported race to label three ethnic groups within the ADNI samples, Black/African-American (ADNI1), White (ADNI2), or Asian (ADNI3).

### Data preparation and quality control

For the distance-based clustering, the following data preparation steps were taken. First, all palindromic markers (alleles A/T or G/C) were excluded to correct for strand differences between datasets (120,541 markers excluded). We then excluded all markers that were not genotyped in at least two of the three data sets (342,603 markers included). Finally, we removed all markers with genotyping rates lower than 95% across all individuals (263,883 markers included). Individuals with genotyping rates of lower than 95% for the included markers were excluded from the analysis. The final set consisted of 2,274 individuals, 400 from Cache, 626 from ADNI, and 1,248 from the various HapMap populations.

For the model-based clustering, we compared Cache to only two populations (CEU and ADNI2), because they appeared most similar in the distance-based method. This included a total of 1,155 samples. Pruning of markers to minimize redundant information due to linkage disequilibrium was done using the following parameters in PLINK: r^2 ^> .10 in 50 SNP windows, incremented by 5 SNPs across the genome (61616 markers included).

### Methods for distance and model-based clustering analysis

The distance-based method of comparison is a standard classical (metric) multi-dimensional scalar analysis of pairwise identity by state distances. Distances were calculated and scaling was performed using the PLINK whole genome association analysis toolset [7].

In addition to distance scaling, Bayesian modeling techniques were leveraged to identify clustering of samples from Cache and the other European-American populations. The model-based clustering method was provided using the program Structure [8]. Burn-in duration was set at 5,000 repetitions with a run duration of 10,000 repetitions per software recommendations[8]. We used an admixture type ancestry model due to the generally admixed nature of the European American population and specified for a model of correlated allele frequency across result populations. The statistic of interest generated by Structure is a probability, Pr (Z | P, X), where Z is a vector containing the assignment to a cluster of each individual, P specifies the frequency of each allele at each locus (a statistic that is calculated by Structure to characterize each cluster), and × is the given genotypes of the sampled individuals.

## Results

### Results from the distance-based clustering approach

**Figure 1:**
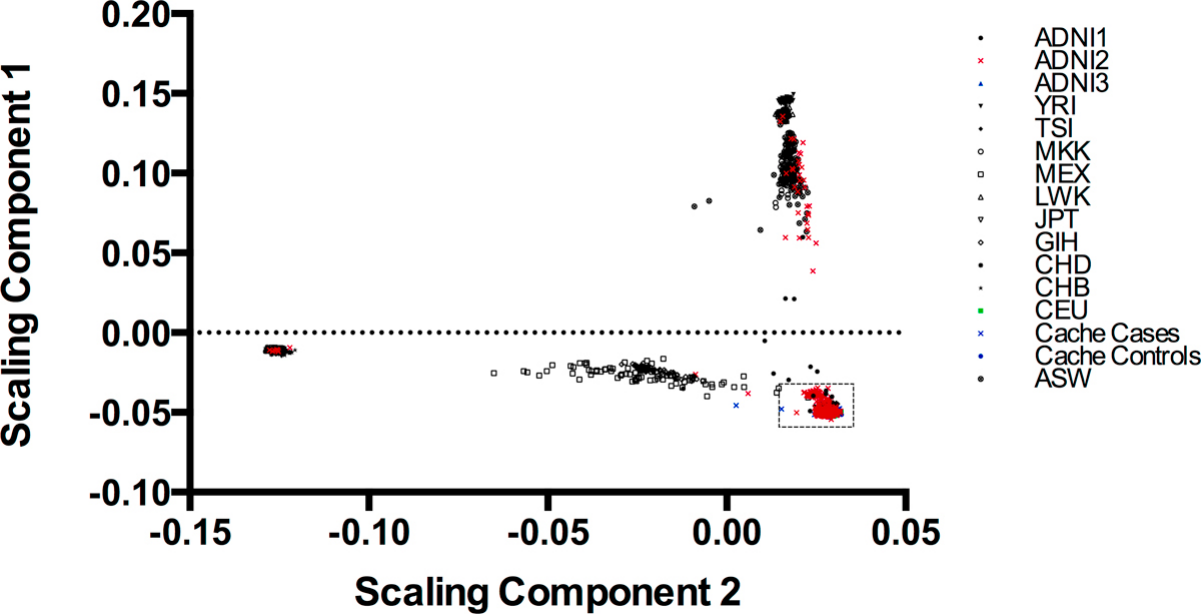
**All individuals with scaling components**. ADNI1 refers to individuals participating in the ADNI study who self-identified as "Black/African-American", ADNI2 refers to those who self-identified as "White", and ADNI 3 refers to those who self-identified as "Asian". All other abbreviations are from Table 1. In Cache samples, case or control status refers to clinical diagnosis of Alzheimer's disease. Dashed box designates the region displayed in figure 2 and includes the ADNI2, CEU, Cache Cases and Controls.

**Figure 2:**
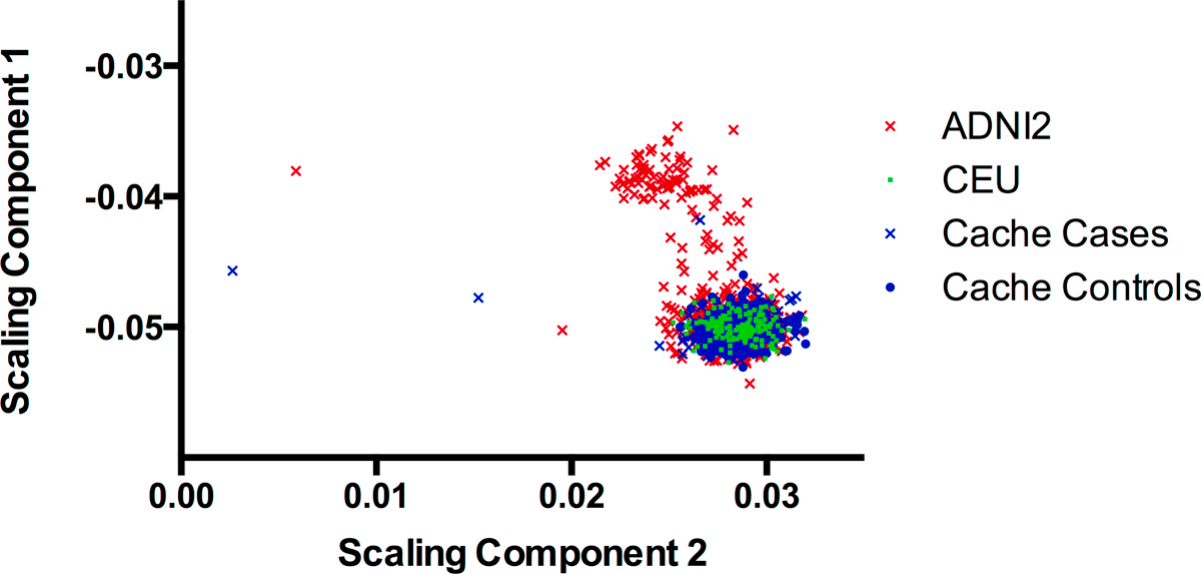
**Detail from Figure 1**. ADNI2 refers to those in ADNI who self reported their ethnicity as "White". CEU are the CEPH families from the HapMap Project and Cache cases and controls are with respect to Alzheimer's disease diagnosis.

Results indicate that samples from the Cache County study cluster very closely with other European American samples (ADNI2 and CEU; Figure 1). In addition, the Cache County samples exhibit a range of diversity that is similar to that of self reported whites from the ADNI sample (ADNI2; Figure 2).

### Results from the model-based clustering approach

Structure results indicate that the observed range of genotypes is most likely to have occurred if the individuals came from two separate populations, as opposed to any other number of populations from one to five (table 2). Additionally, the majority of individuals came from only one of the two proposed clusters (table 3).

**Table 2:** Log of probability of (k) from the combined ADNI2, CEU and Cache County Samples calculated using Structure.

Prior population estimate (k)	ln( P ( Z | P, X) )
1	-73,240,718.0
2	-73,000,731.9
3	-74,033,402.2
4	-87,968,158.6
5	-136,266,205.0

**Table 3:** Cluster assignments from structure.

Sample population	Proportion of individuals assigned to minor result cluster	Proportion of individuals assigned to major result cluster
Cache	.001	.999
ADNI2	.060	.940
CEU	.001	.999

## Discussion

The Cache County study has provided important information about genetic factors that influence aging and Alzheimer's disease [1,9-13]. The results reported here indicate that despite being collected from a single county in northern Utah, these samples are comparable in levels of diversity to those of self-reported European-Americans collected at multiple sites across the United States. In addition, we observed no evidence of problematic population stratification between the Alzheimer's disease cases and non-demented controls in the Cache samples. This indicates that association studies in this population are unlikely to produce type-I error due to population stratification.

The distance-based clustering method identified similarity between the majority of European-American individuals, including those from Cache County, CEU, and ADNI2. The model-base clustering method found the optimum solution to be two clusters, one small cluster with a small number of outliers from each of the three samples (Cache, ADNI2, and CEU) and a larger cluster that included the vast majority of samples.

These data suggest that the population substructure in the Cache County study is comparable to that of other European-American samples that are more broadly collected, (i.e. the ADNI samples). In addition, these findings are consistent with the results reported previously on the larger population of the early population of the state of Utah [2-4]. We conclude that despite concerns about the limited geographic range of sample collection in the Cache County study, the results of genetic studies in this population, such as gene discovery, validation, and estimates of population level effects, are as broadly applicable to other populations of European American ancestry as that of the Alzheimer's Disease Neuroimaging Initiative.

## Competing interests

The authors declare no conflicts of interest or competing interests with regard to this work.

## Declarations

This work was supported by the Alzheimer's Association (MNIRG-11-205368), the BYU Gerontology Program and the National Institutes of Health (R01-AG11380, R01- AG021136, P30-NS069329-01, R01-AG042611). The Institutional Review Boards of each appropriate institution approved sample and data collection.

This article has been published as part of *BMC Bioinformatics *Volume 15 Supplement 7, 2014: Selected articles from the 10th Annual Biotechnology and Bioinformatics Symposium (BIOT 2013). The full contents of the supplement are available online at http://www.biomedcentral.com/bmcbioinformatics/supplements/15/S7
